# Banna Virus, China, 1987–2007

**DOI:** 10.3201/eid1603.091160

**Published:** 2010-03

**Authors:** Hong Liu, Ming-Hua Li, You-Gang Zhai, Wei-Shan Meng, Xiao-Hong Sun, Yu-Xi Cao, Shi-Hong Fu, Huan-Yu Wang, Li-Hong Xu, Qing Tang, Guo-Dong Liang

**Affiliations:** Institute for Viral Disease Control and Prevention, Chinese Center for Disease Control and Prevention, Beijing, People’s Republic of China

**Keywords:** Banna virus, distribution, phylogeny, China, viruses, dispatch

## Abstract

Banna viruses (BAVs) have been isolated from pigs, cattle, ticks, mosquitoes, and human encephalitis patients. We isolated and analyzed 20 BAVs newly isolated in China; this finding extends the distribution of BAVs from tropical zone to north temperate climates and demonstrate regional variations in BAV phylogeny and mosquito species possibly involved in BAV transmission.

Banna virus (BAV), the prototype species of genus *Seadornavirus* within the family *Reoviridae*, has a genome composed of 12 segments of double-stranded RNA (*1*). BAV was initially isolated from persons with encephalitis and fever in Xishuangbanna, Yunnan Province, People’s Republic of China, in 1987 (*2*). Since then, BAV isolates have been obtained from pigs, cattle, and ticks in China (*3**,**4*) and from mosquitoes in Indonesia, China, and Vietnam. (*5**–**7*). BAV is a BioSafety Level 3 arboviral agent that is pathogenic to humans and may well be an emerging pathogen or undiagnosed cause of human viral encephalitis in some areas (*1*). Our objective was to describe new BAV isolates from China and to define the geographic distribution and the phylogenetic relationships of these isolates with reference to the previously described isolates.

## The Study

**Figure 1:**
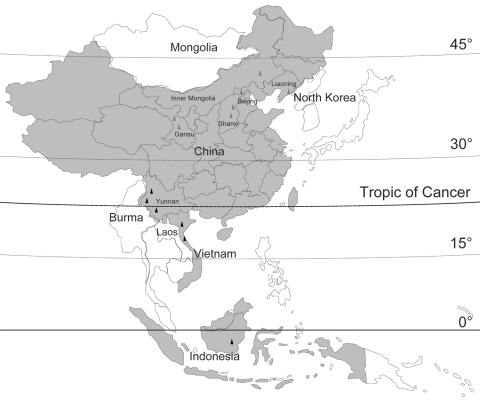
Location of new Banna viruses (BAVs) isolated in China (red triangles) and previously reported BAV isolation sites (black triangles). Countries reporting isolation of BAV are shaded. The names of the countries that are contiguous with BAV isolation sites are labeled. BAV distribution sites in Indonesia, Vietnam, and part of China are located in tropical zones, which lie predominantly between the Tropic of Cancer and the equator. Most BAV distribution sites in China in the area from the Tropic of Cancer to latitude 45°N belong to the northern temperate zone.

In this study, 20 new BAV isolates were obtained from mosquitoes collected from July through September during 2006 to 2007 at sites in Gansu Province (latitude 32°–35°N, 104°–107°E), Liaoning Province (39°–41°N, 123°–125°E), Shanxi Province (37°–38°N, 111°–113°E), and Inner Mongolia Province (41°–43°N, 121°–123°E) (Table, Figure 1). Mosquito samples were collected by using 12 V, 200 mA mosquito-trapping lamps (Wuhan Lucky Star Environmental Protection Tech Co., Ltd., Hubei, China) and by collecting mosquitoes from 8:00 pm to 11:00 pm at nearby cow barns, a piggery, and fish pond sites where human activity was frequent. Mosquitoes were put into a –20°C freezer for 30 min and then were rapidly sorted into pools of 50 to 100 specimens according to species. The pools were put into labeled tubes and stored in liquid nitrogen.

**Table:** Distribution of Banna viruses in regions and vectors, China

Region	Country	Province	Strain	Origin	Date of collection	Vector	Accession no.	Reference
Temperate zone	China	Gansu	GS07-KD12	Cow barn	2007 Aug	*Anopheles sinensis*	GQ331954	This study
			GS07-KD15	Cow barn	2007 Aug	*Culex tritaeniorhynchus*	GQ331955	This study
			GS07-KD16	Cow barn	2007 Aug	*Cx. pipiens pallens*	GQ331956	This study
			GS07-KD18	Cow barn	2007 Aug	*An. sinensis*	GQ331957	This study
			GS07-KD27	Piggery	2007 Aug	*Cx. tritaeniorhychus*	GQ331958	This study
			GS07-KD29	Piggery	2007 Aug	*Aedes albopictus*	GQ331959	This study
			GS07-KD30	Piggery	2007 Aug	*Cx. pipiens pallens*	GQ331960	This study
			GS07-KD32	Piggery	2007 Aug	*Cx. pipiens pallens*	GQ331961	This study
			GS07-KD38	Piggery	2007 Aug	*Cx. pipiens pallens*	GQ331962	This study
			GS-KD42-2	Piggery	2006 Aug	*Cx. tritaeniorhychus*	FJ160414	(*8*)
		Shanxi	SX0765	Piggery	2007 Aug	*Cx. pipiens pallens*	GQ331963	This study
			SX0766	Piggery	2007 Aug	*Cx. pipiens pallens*	GQ331964	This study
			SX0767	Piggery	2007 Aug	*Ae. vexans*	GQ331965	This study
			SX0771	Piggery	2007 Aug	*Cx. pipiens pallens*	GQ331966	This study
			SX0789	Piggery	2007 Aug	*Ae. dorsalis*	GQ331967	This study
			SX0790	Piggery	2007 Aug	*Ae. vexans*	GQ331968	This study
			SX0793	Piggery	2007 Aug	*Cx. pipiens pallens*	GQ331969	This study
			SX0794	Piggery	2007 Aug	*Ae. dorsalis*	GQ331970	This study
			SX0795	Piggery	2007 Aug	*Cx. pipiens pallens*	GQ331971	This study
			SX0796	Piggery	2007 Aug	*Cx. pipiens pallens*	GQ331972	This study
		Inner Mongolia	NM0706	Fishpond	2007 Aug	*Cx. modestus*	GQ331973	This study
		Liaoning	LN0684	Piggery	2006 Aug	*An. sinensis*	FJ217989	(*11*)
			LN0688	Piggery	2006 Aug	*An. sinensis*	FJ217990	(*11*)
			LN0689	Piggery	2006 Aug	*An. sinensis*	FJ217991	(*11*)
		Beijing	BJ95-75	Unknown	1995	Unidentified mosquito	AY568289	(*12*)
Subtropical zone	China	Yunnan	YN-6	Unknown	2001	Unidentified mosquito	AY568290	(*12*)
			YN0556	Unknown	2005 Jul	*Cx. tritaeniorhychus*	FJ161966	(*10*)
			YN0558	Unknown	2005 Jul	*Cx. tritaeniorhychus*	FJ161964	(*10*)
			YN0659	Unknown	2005 Jul	*An. sinensis*	FJ161965	(*10*)
	Vietnam	Quang Binh	02VN180b	Unknown	2002 Aug	*Cx. tritaeniorhychus*	EU265727	(*7*)
			02VN178b	Unknown	2002 Aug	*Cx. tritaeniorhychus*	EU265715	(*7*)
			02VN018b	Unknown	2002 Mar	*Cx. annulus*	EU265694	(*7*)
		Ha Tay	02VN009b	Unknown	2002 Jan	*Cx. annulus*	EU265682	(*7*)
			02VN078b	Unknown	2002 May	*Cx. tritaeniorhychus*	EU265705	(*7*)
Tropical zone	Indonesia	Java	JKT-6423	Unknown	1980	*Cx. pseudovishnui*	NC004198	(*5*)
			JKT-6969	Unknown	1981	*Ae. vagus*	AF052008	(*5*)
			JKT-7043	Unknown	1981	*Cx. pipiens pallens*	AF052024	(*5*)

Viruses were isolated and BAV isolates were identified using described procedures (*8*). Trizol reagent category no. 10296-028 (Invitrogen, Carlsbad, CA, USA) was used to extract total RNA. cDNA was prepared by using Ready-to-Go You-Prime First-Strand Beads Kit (Amersham Pharmacia Biotech, Piscatawy, NJ, USA) according to the manufacturer’s protocol. An 850-bp gene fragment from the 12th segment, which codes for the double-stranded RNA binding protein, was amplified from the cDNA of the BAV isolates by using previously published primers (*9*). PCR products were recovered by using purification kits (QIAGEN, Valencia, CA, USA), and then were inserted into pGEM-T easy vector (Promega, Madison, WI, USA). The insert sequence was determined by using M13 universal primers and an ABI Prism 3730 sequence analyzer (ABI, Shirley, NY, USA).

The genomic sequences of the 12th segment for the 20 new BAV strains were determined (GenBank accession nos. GQ331954–GQ331973). Phylogenetic trees were constructed from the amplified region of the 12th segment sequence by using the molecular evolutionary genetics analysis (MEGA) version 4 software (www.megasoftware.net) from aligned nucleotide sequences. We used neighbor-joining algorithms with 1,000 replicates for bootstrap support of tree groupings.

In this study, 38 BAV strains isolated during 1987–2007 were analyzed, which included 30 strains isolated in China (including 20 new BAV isolates first reported in this study and 10 previously described isolates from China (*8**,**10**–**12*), 3 strains from Indonesia, and 5 strains from Vietnam) (Table). Initial BAVs were isolated from Indonesia and Yunnan Province of China, which belong to tropical and subtropical zones (*2**,**5*).The new BAV isolates in our study were observed in Gansu, Shanxi, Liaoning, and Inner Mongolia provinces of China (northern China), which belong to the northern temperate zone. These strains represent a geographic distribution ranging from near the equator to latitude 45°N, extending from the tropical zone to the northern temperate zone (Figure 1). These data show that the distribution of BAVs is not limited to Southeast Asia but that it extends into northeast Asia as well.

Before our study, BAV had been isolated from 7 mosquito species in 2 genera (*Culex tritaeniorhynchus, Cx. pipiens pallens, Cx. annulus, Cx. pseudovishnui, Cx. modestus, Anopheles sinensis,* and *Aedes vagus*). To this list we now add 3 species in the genus *Aedes* (*Ae. albopictus, Ae. vexans,* and *Ae. dorsalis*) (Table), which are widely distributed in China and elsewhere.

**Figure 2:**
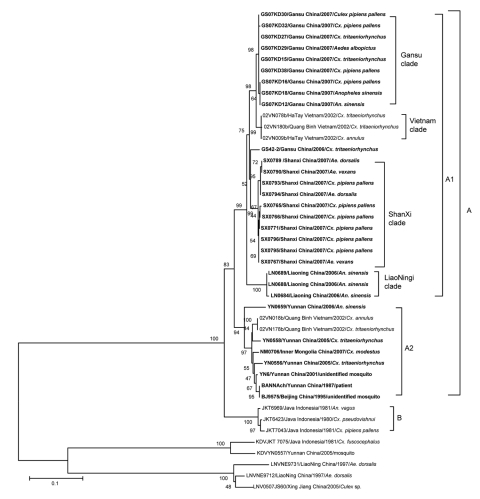
Phylogenetic analysis based on the complete coding sequence of the 12th segment of Banna viruses (BAVs) currently isolated. Phylogenetic analyses were performed by the neighbor-joining method using MEGA version 4 software (www.megasoftware.net). Bootstrap probabilities of each node were calculated with 1,000 replicates. The tree was rooted by using Kadipiro virus and Liaoning virus as the outgroup viruses. Scale bars indicate a genetic distance of 0.1-nt substitutions per site. Isolates obtained in China are in **boldface**. Viruses were identified by using the nomenclature of virus strain/country/year of isolation/origin.

Phylogenetic analysis based on the complete coding sequence (624 nt) of the 12th segment of the BAV genome indicated that the BAV isolates evaluated in this study could be divided into 2 phylogenetically different groups (Figure 2). Isolates from China and Vietnam are included in group A, and the strains from Indonesia are in group B. Group A could be further divided into 2 subgroups, A1 and A2. Subgroup A1 includes 4 independent clades that group according to the location of collection and represent viruses from northern China (Gansu, Shanxi, and Liaoning Provinces) as well as the Vietnam isolates. Subgroup A2 includes isolates mainly from southern China (Yunnan Province) and Vietnam, which is contiguous with Yunnan Province of China, as well as 2 isolates from northern China (BJ95-75/Beijing, and NM0706/Inner Mongolia) (Figure 1).

## Conclusions

Our results demonstrate that BAV strains are distributed from the tropics of Southeast Asia to the northern temperate regions of China. These observations suggest that the distribution of BAV is wider than previously recognized and may be increasing. Consistent with previous observations (*9*), we report that BAV isolates from China cluster in group A and separate into subgroups mainly according to the geographic origin of the isolate; subgroup A1 is found in the north and subgroup A2 in the south. However, 2 isolates from northern China grouped in subgroup A2 (south), and 3 isolates from Vietnam grouped in subgroup A1 (north).

Considering that group A isolates are geographically located across the monsoon climate zone, where south-to-north winds are common during summer (*13*), BAV could be transferred in infected mosquitoes during this period by the prevailing winds that move from Southeast Asia to east Asia. In addition, bird migration, has been associated with the movement of other pathogens, and migration of infected birds through the east Asia–Australasia flyway (*13*), which traverses the region, may also account for this association. However, the transmission dynamics of BAVs are not well known. Further study is required to determine if winds and birds are involved in dispersal of the virus.

Our observations suggest that the public health impact of BAV may be underestimated. BAV appears to be actively circulating in areas where Japanese encephalitis virus (JEV) is endemic (*14*) and where *C. tritaeniorhynchus*, which is the main vector of JEV, is active. This mosquito also appears to be a common vector of BAV. The clinical symptoms of disease caused by the 2 viruses is similar, and BAV cases may be undetected during a JE outbreak. It has been reported that ≈14% of clinically diagnosed JE cases are BAV immunoglobulin (Ig) M positive (*15*), indicating that BAV epidemics may have occurred but have been clinically misdiagnosed as Japanese encephalitis. The apparent active transmission of BAV over a large geographic area, genetic variation between geographic regions, and the potential to cause severe disease underscore the need for additional surveillance, further characterization, and improved diagnostic systems worldwide.

## References

[1] Attoui H, Mohd Jaafar F, Micco P, Lamballerie X. Coltiviruses and seadornaviruses in North America, Europe, and Asia. Emerg Infect Dis. 2005;11:1673–9. 16318717 10.3201/eid1111.050868 PMC3367365

[2] Xu P, Wang Y, Zuo J, Lin J, Xu PM. New orbiviruses isolated from patients with unknown fever and encephalitis in Yunnan province [in Chinese]. Chin J Virol. 1990;6:27–33.

[3] Xu P, Wang Y, Zuo J, Lin J. Recovery of the same type of virus as human new orbivirus [in Chinese]. Chin J Virol. 1990;6:328–32.

[4] Li QP, Xe XC. First isolation of new orbivirus (Banna) from ticks and infected cattle sera in Xingjiang [in Chinese]. Endemic Dis Bull. 1992;7:64–9.

[5] Brown SE, Gorman M, Tesh B, Knudson L. Coltiviruses isolated from mosquitoes collected in Indonesia. Virology. 1993;196:363–7. 10.1006/viro.1993.1490 8102827

[6] Chen B, Tao SJ. Arboviruses survey in China in recent ten years. Chin Med J (Engl). 1996;109:13–5. 8758350

[7] Nabeshima T, Thi Nga P, Guillermo P, Parquet MC, Yu F, Thanh TN, Isolation and molecular characterization of Banna virus from mosquitoes, Vietnam. Emerg Infect Dis. 2008;14:1276–9. 10.3201/eid1408.080100 18680655 PMC2600385

[8] Zhai YG, Wang HQ, Xu HK, Meng WS, Chao YX, Fu SH, Investigation on arboviruses in Tianshui and Longnan regions of Gansu Province [in Chinese]. Chin J Zoonoses. 2008;24:59–63.

[9] Billoir F, Attoui H, Simon S, Gallian P, Micco P, Lamballerie X. Molecular diagnosis of group B coltiviruses infections. J Virol Methods. 1999;81:39–45. 10.1016/S0166-0934(99)00055-5 10488759

[10] Sun X, Fu S, Gong Z, Ge J, Meng W, Feng Y, Distribution of arboviruses and mosquitoes in northwestern Yunnan Province, China. Vector Borne Zoonotic Dis. 2009; 19196130 10.1089/vbz.2008.0145

[11] Meng WS, Zhang JB, Sun XH, Liu QN, Chen Z, Zhai YG, Isolation and identification of arboviruses from mosquito pools in some regions of Liaoning Province, China [in Chinese]. Chin J Epidemiol. 2009;30:50–4. 19565849

[12] Xu LH, Tao SJ, Cao YX. Genotyping of the Chinese isolation of coltivirus [in Chinese]. Chin J Virol. 2003;17:346–9.

[13] Nga PT, del Carmen Parquet M, Cuong D, Ma P, Hasebe F, Inoue S, Shift in Japanese encephalitis virus (JEV) genotype circulating in northern Vietnam: implications for frequent introduction of JEV from Southeast Asia to East Asia. J Gen Virol. 2004;85:1625–31. 10.1099/vir.0.79797-0 15166447

[14] Erlanger TE, Weiss S, Keiser J, Utzinger J, Wiedenmayer K. Past, present, and future of Japanese encephalitis. Emerg Infect Dis. 2009;15:1–7. 10.3201/eid1501.080311 19116041 PMC2660690

[15] Tao SJ, Chen BQ. Studies of coltivirus in China. Chin Med J (Engl). 2005;118:581–6. 15820089

